# Enhanced Proton Conductivity Promoted by Structural Transition in a 2D Interwoven Metal–Organic Framework

**DOI:** 10.1021/acs.cgd.4c01279

**Published:** 2024-12-24

**Authors:** Xi Chen, Nippich Kaeosamut, Sergei Sapchenko, Xue Han, Qingqing Mei, Ming Li, Inigo J. Vitorica-Yrezabal, Lewis Hughes, Sihai Yang, Martin Schröder

**Affiliations:** † Department of Chemistry, University of Manchester, Manchester M13 9PL, U.K.; ‡ College of Chemistry Beijing Normal University, Beijing 100875, China; § School of Engineering, University of Nottingham, Nottingham NG7 2RD, U.K.; ∥ Department of Earth and Environmental Sciences, The University of Manchester, Manchester M13 9PL, U.K.; ⊥ Beijing National Laboratory for Molecular Sciences, College of Chemistry and Molecular Engineering, Peking University, Beijing 100871, China

## Abstract

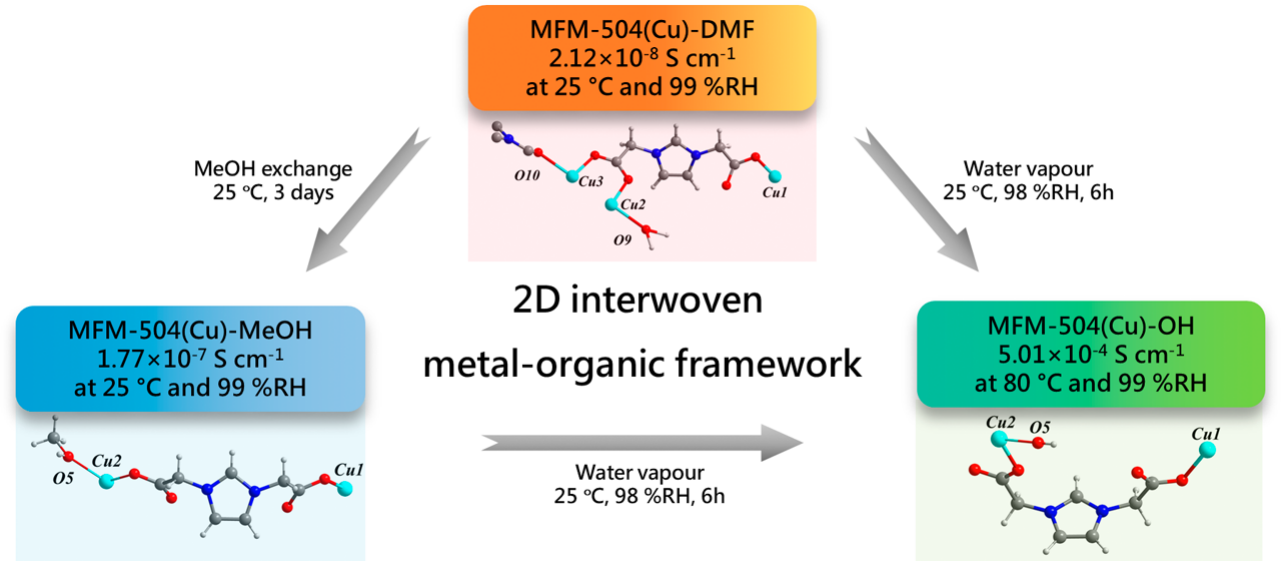

We report enhanced proton conductivity promoted by a structural phase transition of MFM-504(Cu)-DMF to MFM-504(Cu)-MeOH and to MFM-504(Cu)-OH via ligand substitution upon exposure to MeOH and H_2_O vapors, respectively. MFM-504(Cu)-DMF can be synthesized by the solvothermal reaction of Cu(NO_3_)_2_·3H_2_O and the flexible zwitterionic ligand, imidazolium-1,3-bis(methylenedicarboxylate) (imidc^–^), to afford a unique layered interwoven network structure. MFM-504(Cu)-OH shows a proton conductivity of 5.01 × 10^–4^ S cm^–1^ at 80 °C and 99% relative humidity (RH) with an activation energy (*E*_a_) of 0.80 eV, indicating a vehicle mechanism within MFM-504(Cu)-OH.

## Abstract 2

Proton conductivity is promoted by a structural phase transition of MFM-504(Cu)-DMF to MFM-504(Cu)-MeOH and to MFM-504(Cu)-OH via ligand substitution upon exposure to MeOH and H_2_O vapors, respectively.

## Introduction

Metal–organic frameworks (MOFs) are porous crystalline materials that show rich structural diversity due to their tunable porosity and functionality^1^ and have been studied for a wide range of applications, such as gas storage and separation,^2,3^ catalysis,^4^ supercapacitors,^5^ and sensing.^6^ In recent years, MOFs with high proton conductivity (>10^–2^ S cm^–1^) have emerged as candidates for applications in proton exchange membrane fuel cells.^7−14^ Functionalization of the organic ligands with acidic groups (e.g., –SO_3_H,^7^ –PO_3_H_2_,^8^ –COOH^9^) and adsorption of guest molecules with intrinsic proton conductivity [e.g., H_2_SO_4_,^10^ H_3_PO_4_,^11^ ionic liquids (ILs)^12,13^] have been adopted to enhance the proton conductivity of MOFs. The involvement of structural phase transitions has been used as a novel strategy to adjust the proton conductivity both in porous and nonporous MOFs.^7,15,16^ For example, BUT-8(Cr)A shows an increase in the proton conductivity from 6.32 × 10^–3^ S cm^–1^ [25 °C and 65% relative humidity (RH)] to 7.61 × 10^–2^ S cm^–1^ (25 °C under 100% RH), linked to a structural transition on adsorption of H_2_O molecules into the pores.^7^ Recently, MFM-722(Pb)-H_2_O has been shown to exhibit a proton conductivity of 1.33 × 10^–4^ S cm^–1^ (25 °C and 99% RH) after a single-crystal-to-single-crystal (SCSC) structural transformation from an original value of 8.09 × 10^–5^ S cm^–1^.^15^

Ionic liquids (ILs) are a class of solvents that can replace organic solvents due to their low vapor pressure, nonflammability, and high chemical stability, and have thus been explored for the synthesis of MOFs.^17−19^ Moreover, ILs can act as linkers to bridge metal ions directly.^20^ Imidazolium-1,3-bis(methylenedicarboxylate) (imdc^–^)^21^ is an IL-based linker that has two carboxylate groups available to bridge metal ions, balanced by a cationic charge on the imidazolium moiety. To date, several MOFs have been reported with this linker and its derivatives, and these are noted by the conformational freedom at the –CH_2_– group between imidazolium and carboxylate groups.^22−26^ Most of these are 2-fold interpenetrated three-dimensional (3D) structures, making them promising candidates for catalysis and fluorescence sensing.^25,26^ However, their proton conductivity has been poorly explored.^13^ Here, we report the structural phase transition via ligand substitution in a flexible IL-based MOF, MFM-504(Cu)-DMF, and the enhancement of proton conductivity in the resultant derivative MFM-504(Cu)-OH. Besides the conformational freedom of the –CH_2_– group of the linker, the flexible coordination environment at distorted Cu(II) centers affords the possibility of structural diversity. In situ impedance spectroscopy has been employed to evaluate the change in proton conductivity of MFM-504(Cu)-DMF during a structural phase transition on exposure to H_2_O vapor, and analysis of the crystal structure reveals a hydrogen bonding network that supports proton transfer.

## Results and Discussion

**Figure 1:**
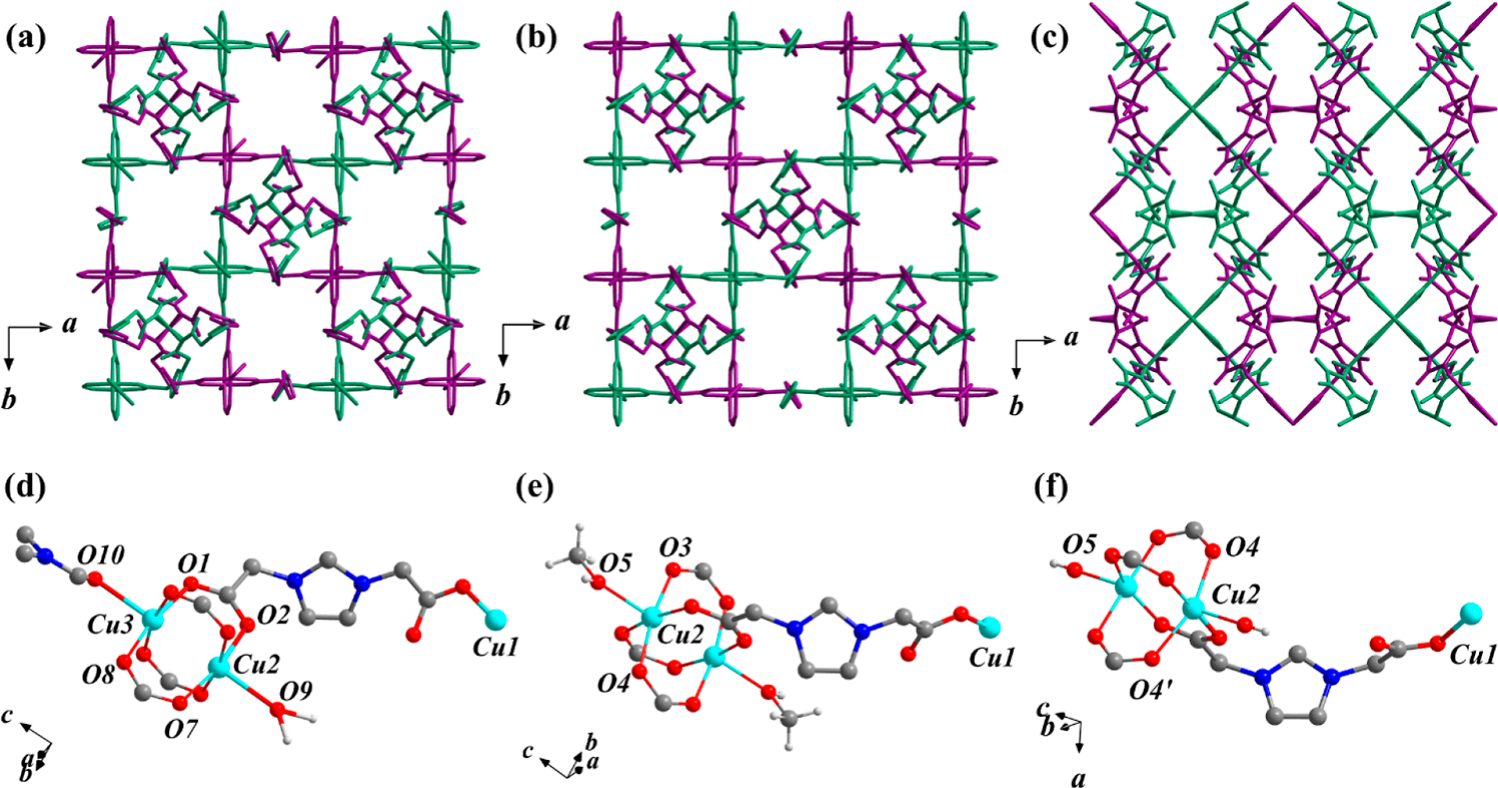
Views of the 2D layered structures of (a) MFM-504(Cu)-DMF, (b) MFM-504(Cu)-MeOH, and (c) MFM-504(Cu)-OH along the *c* axis. Two interwoven networks are shown in green and purple. Hydrogen atoms are omitted for clarity. Views of the metal–ligand coordination on (d) MFM-504(Cu)-DMF, (e) MFM-504(Cu)-MeOH, and (f) MFM-504(Cu)-OH (Cu: cyan, O: red, N: dark blue, C: dark gray H: light gray).

MFM-504(Cu)-DMF was synthesized by the solvothermal reaction of Cu(NO_3_)_2_·3H_2_O and H_2_imdc·Br in DMF at 55 °C for 10 h, and isolated as green rod-shaped single crystals. Single-crystal X-ray diffraction reveals that MFM-504(Cu)-DMF, [Cu_3_(imdc)_4_(DMF)_0.5_(H_2_O)_1.5_]·(OH)_2_, crystallizes in the tetragonal space group *P*4̅2_1_*m*, showing a 2D interwoven network along the *c* axis (Figure 1a). This yields a 1D channel of 3 × 4 Å running along the *c* axis, which is partially occupied by coordinated DMF and water molecules (Figure S8a). The corresponding coordination environment of the ligand is shown in Figure 1d. O1 and O2 from one of the carboxylate groups bind to Cu2 and Cu3 in the [Cu_2_(O_2_CR)_4_] paddlewheel in a bidentate mode, and O4 from another carboxylate group coordinates to Cu1 in a monodentate mode. There are three crystallographically independent Cu(II) ions (Figures 1d and S9a), two of which are 5 coordinate to O donors and form the [Cu_2_(O_2_CR)_4_] paddlewheel [Cu···Cu = 2.66(1) Å, ∠O2Cu2O7 = 168.1(3)°, ∠O1Cu3O8 = 169.0(3)°]. O9 and O10 are from water and DMF molecules, respectively. The bond length for Cu2–O9(H_2_O) of 2.35(2) Å consistent with the Cu–O(H_2_O) bond lengths reported for HKUST-1 [2.156(1) Å to 2.207(5) Å].^27,28^ In addition, the reported bond lengths for Cu–O(OH−) are typically in the range of 1.929(5) to 2.153(4) Å.^29^ On this basis, we assign H_2_O molecules as binding to Cu2 in MFM-504(Cu)-DMF rather than OH^–^. Cu1 has a distorted square planar coordinate geometry (Figure S9a) [Cu1···O5 = 1.96(1) Å, Cu1···O4 = 1.95(1) Å, ∠O4Cu1O5 = 87.9(3)°]. Two chains of [Cu_3_(imdc)_4_]_∞_^2+^ are linked through Cu1 to form 2D layers in the *bc* plane (Figure S10). The weaving geometry through Cu1 of MFM-504(Cu)-DMF is similar to that observed in a 2D woven fiber connected through Fe(II) ions.^30^ Powder X-ray diffraction (PXRD) of MFM-504(Cu)-DMF confirms its phase purity (Figure S2), and thermogravimetric analysis (TGA) confirms that the solvent molecules can be removed at 30–90 °C (weight loss of 6%, calcd 6%) followed by a framework decomposition at ∼190 °C (Figure S3).

By immersing single crystals of MFM-504(Cu)-DMF in MeOH for 3 days at 25 °C (Scheme S2), we observed a single crystal-to-single crystal transformation via ligand substitution to give MFM-504(Cu)-MeOH, [Cu_3_(imdc)_4_(MeOH)(H_2_O)]·(OH)_2_. MFM-504(Cu)-MeOH crystallizes in the tetragonal space group *P*4_2_/*ncm*, in which the layered structure and coordination environment of the ligand are similar to those observed for MFM-504(Cu)-DMF (Figure 1b,e). On substitution, O5 from MeOH, with an occupancy of 0.5 is coordinated to Cu2 in the [Cu_2_(O_2_CR)_4_] paddlewheel [Cu···Cu = 2.66(1) Å, ∠O3Cu2O4 = 168.4(3)°] (Figure 1e). O5 from H_2_O (occupancy = 0.5) is also coordinated to Cu2. The bond length of Cu2–O5(H_2_O) is 2.28(8) Å, similar to that observed for Cu2–O9(H_2_O) in MFM-504(Cu)-DMF [2.35(2) Å], providing further evidence that H_2_O is bound to Cu2 over the OH^–^. The coordination environment of Cu1 shows only minor changes [Cu1···O1 = 1.94(1) Å, ∠O1Cu1O1′ = 87.2(3)°] (Figure S9b) and affords a 1D channel of 4 × 4 Å along the *c* axis (Figure S8b).

**Figure 2:**
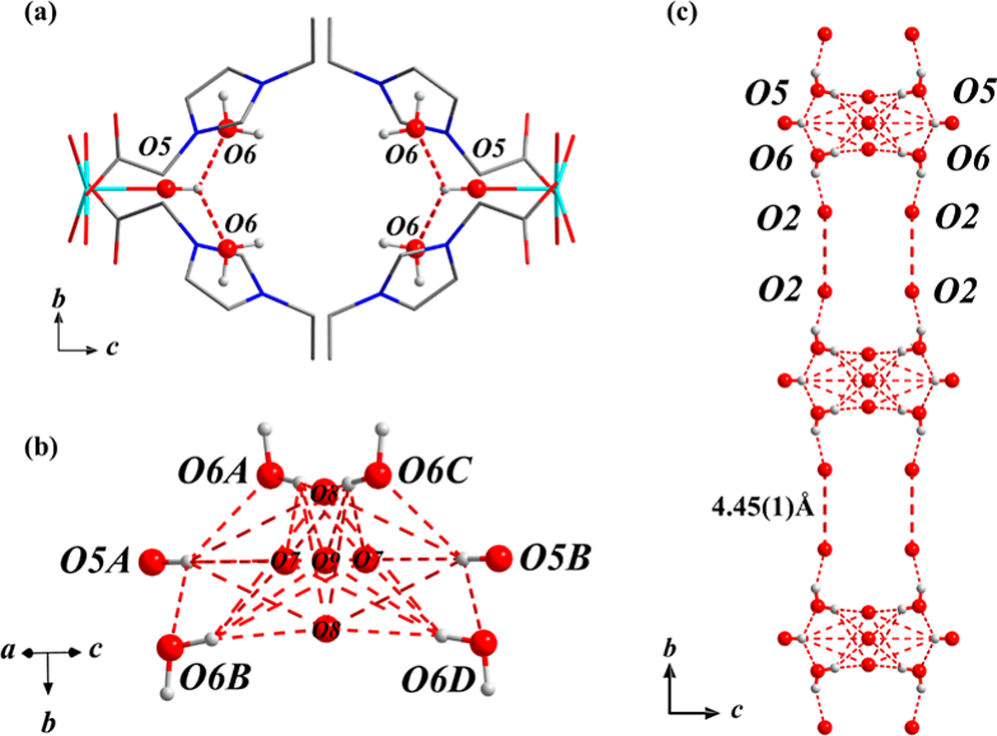
Views of the hydrogen-bonding network in MFM-504(Cu)-OH. (a) Hydrogen bonds O5–H···O6 between the hydroxyl group and the water molecule viewed down the *a*-axis; (b) hydrogen-bonded network in the *bc* plane; (c) the extended [O6] supra-polyhedron running along the *b*-axis (Cu: cyan, O: red, N: dark blue H: light gray, hydrogen bond: red dashed line).

On exposure of single crystals of MFM-504(Cu)-DMF to H_2_O vapor (99% RH) for 6 h at 25 °C (Scheme S2), we observed another phase transition via ligand substitution to give MFM-504(Cu)-OH, [Cu_3_(imdc)_4_(OH)_2_]·12H_2_O, the single-crystal structure of which has been reported previously.^25^ MFM-504(Cu)-OH can also be obtained by exposing MFM-504(Cu)-MeOH to water vapor (99% RH) for 6 h at 25 °C (Scheme S2), and PXRD confirms its phase purity (Figure S2). Moreover, the stability of MFM-504(Cu)-OH following proton conductivity measurements was confirmed by PXRD analysis and SEM imaging (Figures S19 and S20, respectively). The crystals prior to proton conductivity measurements appeared larger than those afterwards, suggesting a degree of breakdown, although the overall crystal morphology remained consistent. MFM-504(Cu)-OH crystallizes in the orthorhombic space group *I*222. On ligand substitution, O5 from the hydroxyl groups binds to Cu2 in the [Cu_2_(O_2_CR)_4_] paddlewheel [Cu···Cu = 2.72(1) Å, ∠O3Cu2O4 = 90.4(1)°] (Figure 1f). The bond length for Cu2–O5(OH−) is 2.073(5) Å, notably shorter than that for Cu–O(H_2_O) in both MFM-504(Cu)-DMF [2.35(2) Å] and MFM-504(Cu)-MeOH [2.28(8) Å]. The four-coordinated environment of Cu1′ in MFM-504(Cu)-OH also shows changes [Cu1···O1 = 1.94(1) Å and ∠O1Cu1O1′ = 89.8(1)°; Figure S9c]. The 2D layered structure along the *c* axis is shown in Figure 1c, and the material has an interlayer free space along the *a* axis of dimension 2 × 2 Å (Figures S8c and S11). A close examination reveals that there are 4-fold hydrogen bonds within the pores along the *a* axis in MFM-504(Cu)-OH (Figure 2a) comprising two O5 atoms from the coordinated hydroxyl groups and four O6 atoms from the free water molecules within the pores, O5–H···O6 [O5···O6 = 2.72(1) Å, ∠O5HO6 = 109.0(1)°].

**Figure 3:**
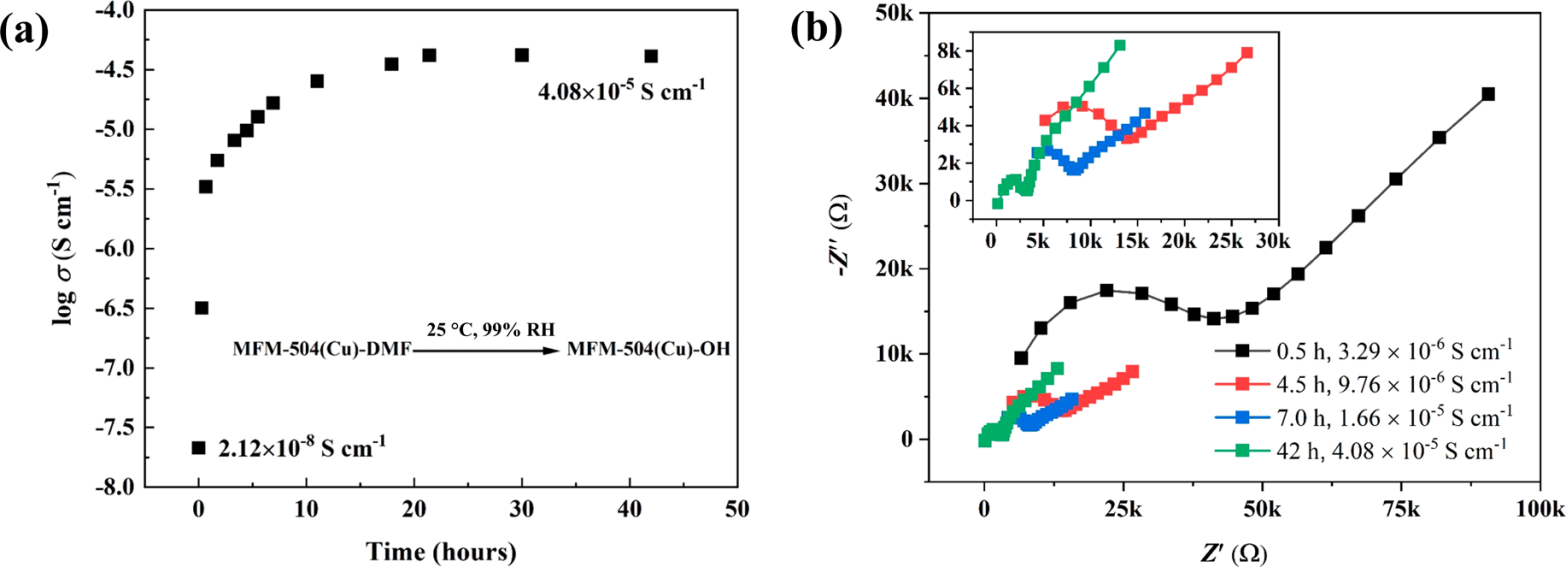
Impedance spectra and proton conductivity of MFM-504(Cu)-DMF and MFM-504(Cu)-OH. (a) Time dependence of the proton conductivity during the phase transition from MFM-504(Cu)-DMF to MFM-504(Cu)-OH at 25 °C and 99% RH; (b) Nyquist plots of MFM-504(Cu)-DMF during the phase transition at various times.

**Figure 4:**
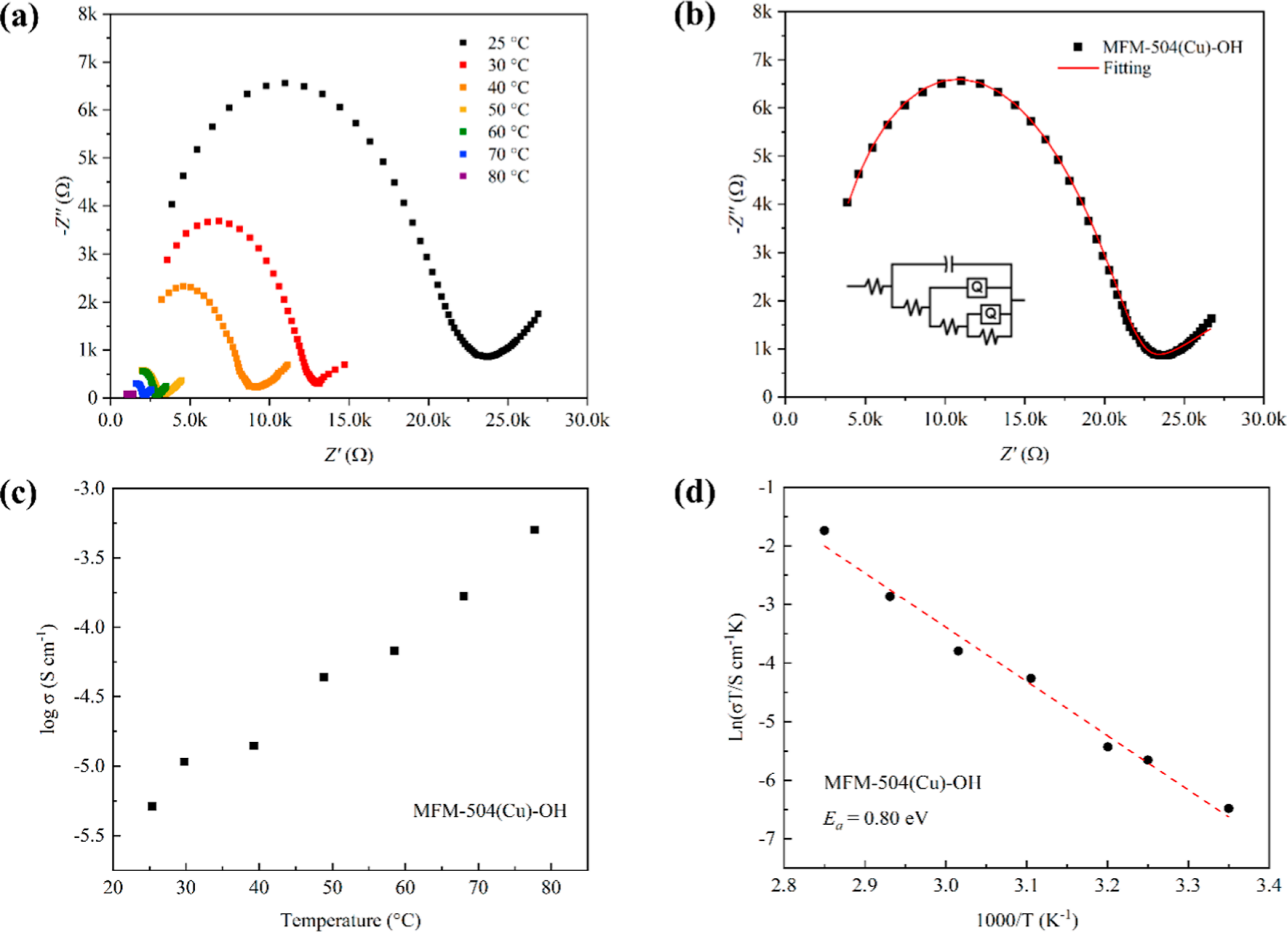
Impedance spectra and proton conductivity of MFM-504(Cu)-OH. (a) Nyquist plot of MFM-504(Cu)-OH at 99% RH at different temperatures; (b) proposed equivalent circuit for fitting of experimental data measured at 99% RH and 25 °C; (c) temperature dependence of the proton conductivity of MFM-504(Cu)-OH at 99% RH at 25–80 °C; (d) Arrhenius plot of the proton conductivity of MFM-504(Cu)-OH at 99% RH.

We sought to monitor in situ the change of proton conductivity of MFM-504(Cu)-DMF during the phase transition using AC impedance spectroscopy (Figure 3). Measurements were carried out on bulk pellets of MFM-504(Cu)-DMF. At 25 °C and 99% RH, the proton conductivity of MFM-504(Cu)-DMF increased gradually from 2.12 × 10^–8^ to 4.08 × 10^–5^ S cm^–1^ over 10 h, and this stabilized over 40 h (Figure 2a). The increase in conductivity originates from the phase transition from MFM-504(Cu)-DMF to MFM-504(Cu)-OH, which is confirmed by PXRD measurements after impedance measurements (Figure S15). Z* plots show a typical semicircle in the high-frequency region indicative of the intrinsic conductivity of the material, and the tail at low frequency represents the blocking of protons at the electrode interface (Figure 3b).^31^ The impedance spectra of MFM-504(Cu)-OH show classical Nyquist plots (Figure 4a), and an example of employing a comparable electrical network for adjusting the empirical data is shown in Figure 4b. Figure 4c shows the temperature dependence of the proton conductivity of MFM-504(Cu)-OH, with the conductivity increasing with increasing temperature reaching 5.01 × 10^–4^ S cm^–1^ at 80 °C and 99% RH. The activation energy (*E*_a_) for MFM-504(Cu)-OH was calculated from the variable-temperature impedance spectra to be 0.80 eV (Figure 4d), suggesting that the proton diffusion is governed by the vehicle mechanism, where the protons are first bound to a carrier and then transported along with the carrier. We also investigated the change of proton conductivity of MFM-504(Cu)-MeOH in situ during the phase transition to MFM-504(Cu)-OH (Figures S16 and S17). The proton conductivity of MFM-504(Cu)-MeOH increased from 1.77 × 10^–7^ to 6.51 × 10^–5^ S cm^–1^ upon phase transition, and PXRD after the impedance measurements confirmed the phase transition (Figure S18).

A detailed examination of the hydrogen bonding network in MFM-504(Cu)-OH was undertaken (Figure 2). Six adjacent oxygen centers (O5A, O5B, O6A, O6B, O6B, and O6D) from two coordinating hydroxyl groups and four water molecules form a [O_6_] supra-polyhedron [O5···O6 = 2.71(1) Å, Table S2] that enables the proton transfer pathway (Figures 2b and S13). This is further supplemented by five additional free water molecules (O7, O8, and O9) inside this suprapolyhedron (Figure S12). The water adsorption isotherm of MFM-504(Cu)-OH shows a total uptake of 7.37 mmol g^–1^ at 20 °C (Figure S6). The number of water molecules adsorbed per metal was calculated as 2.34 molecules. The detailed hydrogen-bonding interactions within the supra-polyhedron in MFM-504(Cu)-OH are illustrated in Figure S13 and Table S2. In addition to the 4-fold strong hydrogen bonds of O5–H···O6 as mentioned above, another four weak hydrogen bonds of O5–H···O7 and O5–H···O8 [O5···O7 = 4.23(1) Å, O5···O8 = 4.24(1) Å] afford an octahedral geometry as shown in Figure S13a. Similarly, O6 can interact with O7 and O8 to form 8-fold hydrogen bonds [O···O = 2.93(1) to 4.06(2) Å; Figure S13b,c, Table S2]. Given the considerable distance between the [O_6_] supra-polyhedron (4.45 Å), it is unlikely that a direct proton transfer pathway occurs between these supra-polyhedron (Figure 2C). Additionally, the activation energy is relatively high, suggesting that water likely functions as the carrier to facilitate proton transfer between the water clusters and, thus, enables proton conductivity.

## Conclusions

In summary, a new flexible 2D layered Cu(II)-IL-based MOF has been synthesized and its framework flexibility structurally characterized. On exposure to MeOH and water, MFM-504(Cu)-DMF undergoes the phase transition to MFM-504(Cu)-MeOH and MFM-504(Cu)-OH, respectively, which is driven by the ligand substitution at room temperature. The proton conductivity of MFM-504(Cu)-OH reaches 5.01 × 10^–4^ S cm^–1^ at 80 °C and 99% RH, 4 orders of magnitude higher than MFM-504(Cu)-DMF. The activation energy (*E*_a_ = 0.80 eV) is consistent with a vehiclular mechanism in which proton carriers act as an intermediary for protont transport. This study will promote the future design of flexible 2D MOFs showing enhanced proton conductivity linked to structural phase transitions.

## Supporting Information Available

Experimental and characterization methods, including PXRD, TGA, IR spectroscopy, adsorption isotherms, crystallographic data, views of structures, and proton conductivity plots and associated data (PDF)
