# Supplementary material for: Enhanced Proton Conductivity Promoted by Structural Transition in a 2D Interwoven Metal–Organic Framework
Source: Cryst Growth Des. 2024 Dec 24;25(2):281–6. doi: 10.1021/acs.cgd.4c01279 (PMC11740994; doi:10.1021/acs.cgd.4c01279)
Supplement: Supplementary file 1 — cg4c01279_si_001.pdf [file cg4c01279_si_001.pdf]

Supporting Information

Enhanced proton conductivity promoted by structural transition in a 2D interwoven metal-organic framework

Xi Chen[1], Nippich Kaeosamut[1], Sergei Sapchenko[1], Xue Han[1.2], Qingqing Mei[1], Ming Li[3], Inigo J. Vitorica-Yrezabal[1], Lewis Hughes[4], Sihai Yang[1,5]* and Martin Schröder[1]*

1 Department of Chemistry, University of Manchester, Manchester, M13 9PL, U.K.
Sihai.Yang@manchester.ac.uk; M.Schroder@manchester.ac.uk

2. College of Chemistry Beijing Normal University, Beijing 100875, China.

3. School of Engineering, University of Nottingham, Nottingham NG7 2RD, U.K.

4. Department of Earth and Environmental Sciences, The University of Manchester, Manchester M13 9PL, U.K.

5. Beijing National Laboratory for Molecular Sciences, College of Chemistry and Molecular Engineering, Peking University, Beijing 100871, China. Sihai.Yang@pku.edu.cn

1. Experimental Section

1.1 Synthesis method

Starting materials were purchased from Acros Organics and Sigma-Aldrich and used without further purification.

Synthesis of 1,3-imidazolium dicarboxylic acid (H_2L) [1]

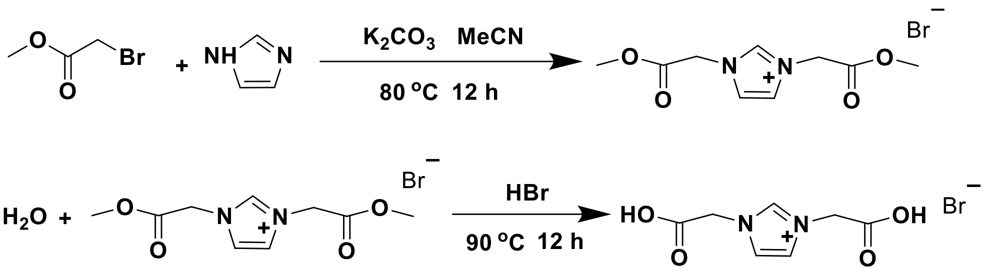

Scheme S1 Synthesis scheme of 1,3-Imidazolium dicarboxylate acid (H_2imdc·Br).

The synthesis of 1,3-imidazolium dicarboxylic acid (H_2imdc·Br) is shown in scheme S1. Imidazole (1.91 g, 0.028 mol) and K_2CO_3 (1.93g, 0.014 mol) were added to CH_3CN (50 mL), and methyl bromoacetate (6 mL, 0.061 mol) added dropwise. The reaction mixture was heated at 80 °C for 12 h in a 100 mL round bottom flask under reflux. The white precipitate of K_2CO_3 was removed by filtration and any remaining CH_3CN and methyl bromoacetate was removed by evaporation to yield a dark brown solution. HBr (40 wt%, 10 mL) was added to the dark brown liquid and heated at 90 °C for 10h to hydrolyze the intermediate. Solvent was removed by rotary evaporation and the solid product collected, washed with EtOH and dried in the oven for overnight. The NMR spectroscopic data for H_2imdc·Br is shown in Figure S1. ^{1}H NMR (300 MHz, D_2O): δ = 5.01 (s, 4H), 7.50 (d, J = 1.6 Hz, 2H), 8.85 (br s, 1H) ppm. The yield is 8.5 g (63.9 %). Elemental analysis and ICP (%): Anal. Calc. for $C_7N_2H_7O_4Br$: C 31.9, H 2.66, N 10.6, Br 30.4; Found: C 32.0, H 3.36, N 10.6, Br 30.3.

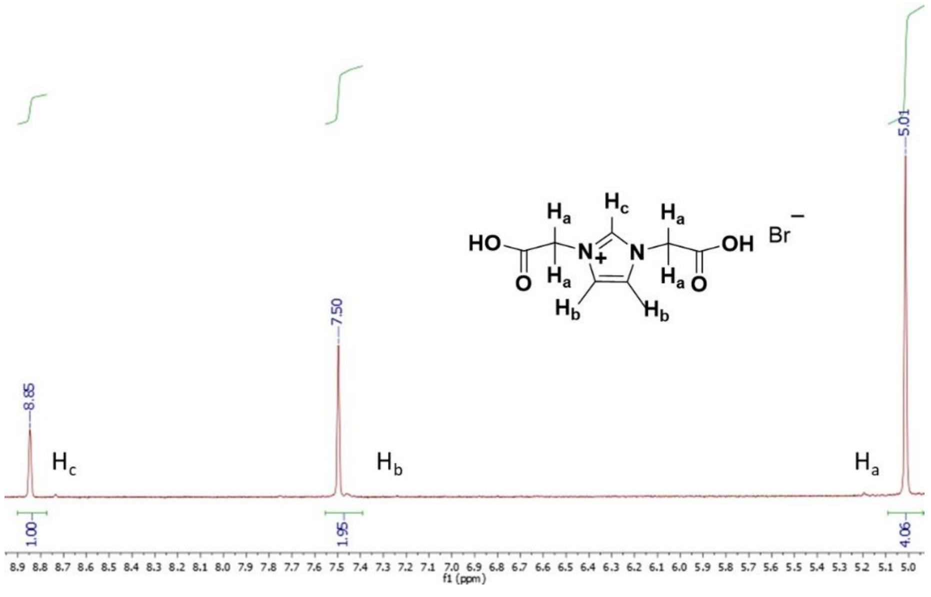

Figure S1. ^{1}H NMR spectrum of 1,3-imidazolium dicarboxylic acid (H_2imdc·Br).

Synthesis of MFM-504(Cu)-DMF [$Cu_3(L)_4(DMF)_{0.5}(H_2O)_{1.5}$]·$(OH)_2$

H_2imdc·Br (0.027 g, 0.10 mmol) and $Cu(NO_3)_2·3H_2O$ (0.048 g, 0.20 mmol) were dissolved in a mixture of DMF (2 mL, N,N-Dimethylformamide), water (0.5 mL) and dilute HNO_3 (0.1 mL, 1mol/L). The mixture was transferred into a pressure vial (8 mL) and heated at 55 °C for 10 h. The green rod single crystals were collected on cooling by filtration and dried in air. Yield: 0.089 g (0.09 mmol), 85 %. Elemental analysis (%): Anal. Calc. for $C_{29.5}H_{35}O_{20}N_{8.5}Cu_3$: C 34.7, H 3.43, N 11.7; Found: C 32.9, H 3.85, N 12.7. ATR-IR for MFM-504(Cu)-DMF (Figure S3): 3158(w), 3088(w), 1663(s), 1384(m), 1297(m), 1182(m), 692(s).

Preparation of MFM-504(Cu)-MeOH [$Cu_3(L)_4(MeOH)(H_2O)$]·$(OH)_2$

Single crystals of MFM-504(Cu)-MeOH were obtained by soaking single crystals of MFM-504(Cu)-DMF in MeOH for 3 days at 25 °C (Scheme S2). Elemental analysis (%): Anal. Calc. for $C_{29}H_{30}O_{20}N_8Cu_3$: C 34.6, H 3.60, N 11.2; Found: C 32.6, H 3.65, N 12.1. ATR-IR for MFM-504(Cu)-MeOH (Figure S3): 3162(w), 3136(w), 3104(w), 1617(s), 1359(m), 1331(m), 1148(s), 716(s).

Preparation of MFM-504(Cu)-OH [$Cu_3(L)_4(OH)_2$]·12(H_2O)

MFM-504(Cu)-OH can be synthesized by soaking the sample of MFM-504(Cu)-DMF in water vapour for 6 h at 25 °C and 99% RH. Elemental analysis (%): Anal. Calc. for $C_{28}H_{54}O_{30}N_8Cu_3$: C 28.7, H 4.60, N 9.60; Found: C 29.5, H 3.24, N 11.3. ATR-IR for MFM-504(Cu)-OH (Figure S3): 3451(w), 1634(s), 1391(m), 1300(m), 1183(m), 997(m), 685(s).

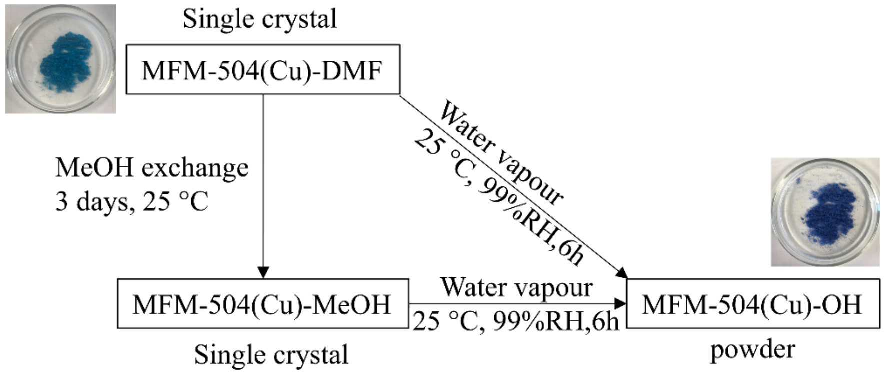

Scheme S2. Routes to products derived from MFM-504(Cu).

1.2 Characterisation methods

X-ray powder diffraction patterns were collected on a Phillips X'pert Pro MPD powder diffractometer at room temperature. ATR-IR spectra were collected on a Thermo Scientific Nicolet iS5-IR spectrometer in the range from 4000 to 400 cm^{-1}, and TGA plots were collected on a Perkin Elmer Pyris1 thermogravimetric analyser under N_2 at a flow rate of 100 mL/min and heating rate of 5 °C /min. Elemental analyses were performed on a Flash 2000 elemental analyser. Scanning electron microscopy (SEM) imaging analysis was performed using an FEI/Thermofisher Quanta 650 field emission gun SEM at the University of Manchester. Water adsorption/desorption isotherms were performed on Autosorb iQ Analyser at 293 K.

Single crystal X-ray data were collected at 150 K on a GV1000 Oxford-Rigaku Supernova diffractometer with Mo-Kα (λ = 0.71073Å) and at 100 K using Rigaku FR-X diffractometer with Cu-Kα (λ = 1.5418Å) equipped with a CCD detector and an Oxford Cryosystems liquid N_2 flow system. Data collection, frame integration and data processing were performed using CrysAlisPro program suite.[2] The structure was solved by direct method and refined on F^2 by full-matrix least-squares method in the anisotropic approximation (for non-hydrogen atoms) using Olex2 program package.[3] The positions of hydrogen atoms of organic ligands were calculated geometrically and refined by a riding model. A summary of the crystallographic data and structural determination for all the compounds is provided in Table S1.

Proton conductivity (σ) was measured by a Solartron SI1260 Impedance analyser over a frequency range of 1 Hz to 1 MHz at the amplitude of 100 mV under the DC rest voltage of 0 mV. The Impedance analyser was connected by an electrochemical cell equipped with platinum current collectors, and the temperature and relative humidity inside the cell was measured using a Rotronic HC2-C04 probe. The pellets were prepared by a press machine under 3 tons to give a thickness of 0.56 mm and a diameter of 8 mm. The top surface and bottom surface were both coated silver paste. The activation energy was calculated by using the *Arrhenius* equation as shown in equation S1:

$$\sigma = (\sigma_0/T)\exp[-Ea/(kT)] \qquad \text{.......equation S1}$$

where σ_0, T and K are the pre-exponential factor, temperature in K and Boltzmann constant, respectively.

2. Characterisation

2.1 PXRD

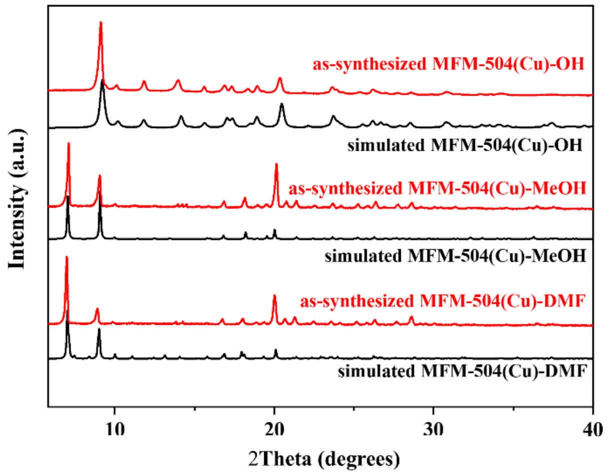

Figure S2. Comparison of the experimental (red) and simulated (black) PXRD patterns of MFM-504(Cu)-DMF, MFM-504(Cu)-MeOH and MFM-504(Cu)-OH.

2.2 TGA

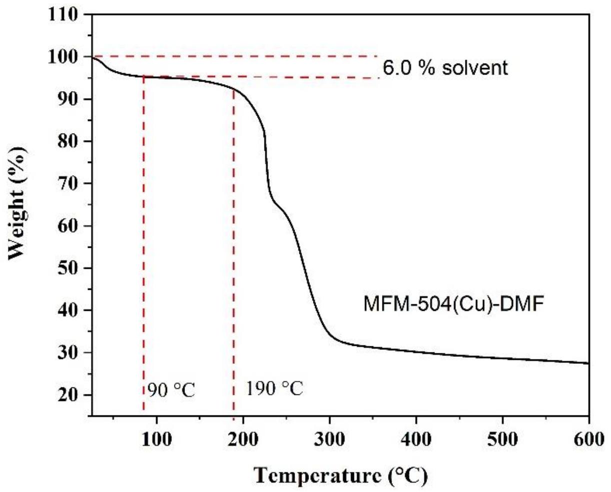

Figure S3. TGA plot of MFM-504(Cu)-DMF.

2.3 FTIR

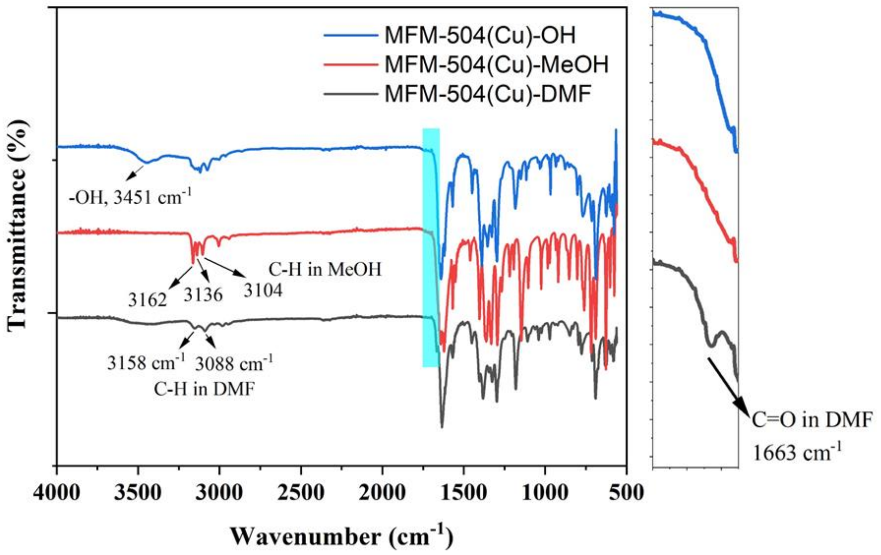

Figure S4. FTIR spectra of MFM-504(Cu)-DMF (black), MFM-504(Cu)-MeOH (red) and MFM-504(Cu)-OH (blue).

The stretching mode of the coordinated hydroxyl groups in MFM-504(Cu)-OH appears at 3451 cm^{-1}. The peaks assigned to the ν(C-H) stretch of methyl groups in DMF are observed at 3158 cm^{-1} and 3088 cm^{-1} in MFM-504(Cu)-DMF. The peaks assigned to the ν(C-H) stretch of methyl groups are observed at 3162 cm^{-1}, 3136 cm^{-1} and 3104 cm^{-1} in MFM-504(Cu)-MeOH. The peak at 1663 cm^{-1} is assigned to the ν(C=O) stretching vibration in DMF molecules, which is absent in both MFM-504(Cu)-OH and MFM-504(Cu)-MeOH.

2.4 N_2 adsorption/desorption isotherms of MFM-504(Cu)-OH

Prior to the BET measurement, the bulk MFM-504(Cu)-OH was activated under vacuum at 120 °C for 12 h. The adsorption-desorption isotherms for N_2 was carried out at 77 K. Figure S5 shows a type-III profile with a surface area of 39.9 m^2 g^{-1}.

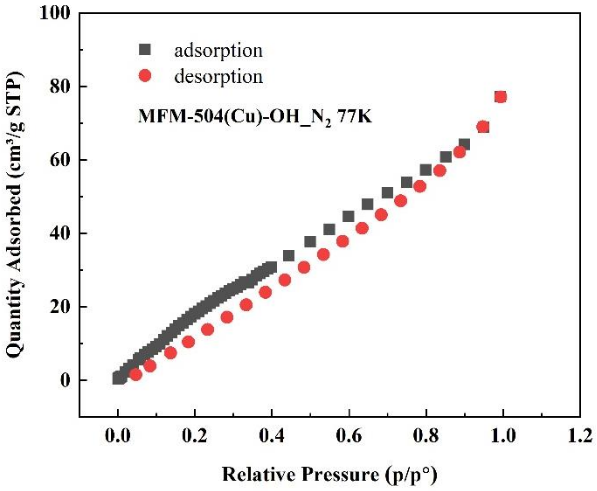

Figure S5. Adsorption and desorption isotherms for N_2 in MFM-504(Cu)-OH at 77 K

2.5 Water adsorption/desorption isotherms of MFM-504(Cu)-OH

MFM-504(Cu)-OH was activated under vacuum at 120 °C for 12 h. Figure S6 shows a total uptake of water of 7.37 mmol g^{-1} at 293K. Despite the PXRD pattern of MFM-504(Cu)-OH after 120ºC activation shows poor crystallinity, crystallinity can be recovered upon water sorption[4] (Figure S7). The initial uptake of water increases linearly with the relative pressure (p/p°=0-0.5), likely due to the adsorption of surface water. At p/p°=0.5, the adsorption curve shows a sharp increase, indicating that the water molecules interact with the MOF through hydrogen bonds to restore the framework crystallinity. The residual water upon desorption further indicates that the water molecules form strong hydrogen-bonding network within the MOF structure.

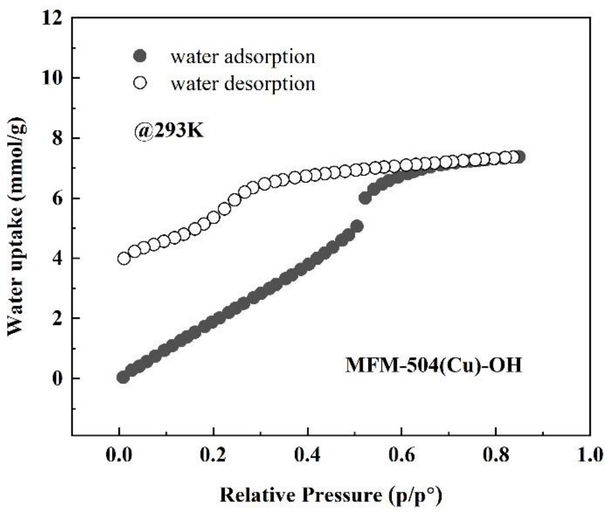

Figure S6. Adsorption and desorption isotherms of water for activated MFM-504(Cu)-OH at 293 K.

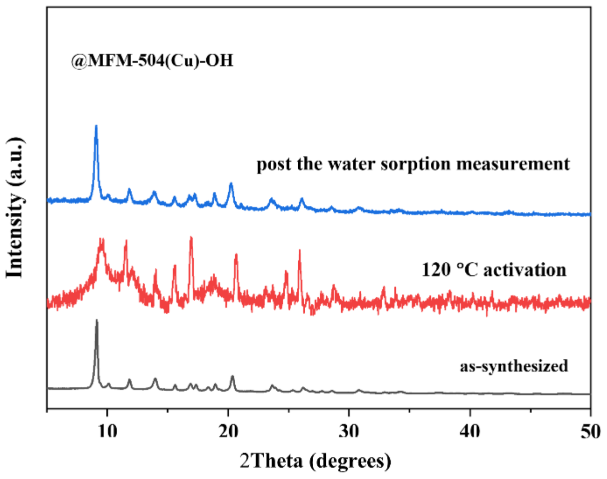

Figure S7. PXRD patterns of as-synthesised MFM-504(Cu)-OH (black), MFM-504(Cu)-OH after 120 °C activation (red) and MFM-504(Cu)-OH post the water sorption measurement (blue).

2.6 Crystallographic data

Table S1. Summary of crystallographic data

Name	MFM-504(Cu)-DMF	MFM-504(Cu)-MeOH	MFM-504(Cu)-OH[a]
Formula	$C_{29.5}H_{35}O_{20}N_{8.5}Cu_3$<br>$[Cu_3(L)_4(DMF)_{0.5}$<br>$(H_2O)_{1.5}]\cdot(OH)_2$	$C_{29}H_{30}O_{20}N_8Cu_3$<br>$[Cu_3(L)_4(MeOH)(H_2O)]$<br>$\cdot(OH)_2$	$C_{28}H_{54}O_{30}N_8Cu_3$<br>$[Cu_3(L)_4(OH)_2]$<br>$\cdot 12(H_2O)$
Radiation	CuKα (λ = 1.5418)	MoKα (λ = 0.7107)	MoKα (λ = 0.7107)
Temperature K	100 K	293 K	173 K
M, g/mol	1019.28	1001.23	1173.40
Crystal system	Tetragonal	Tetragonal	Orthorhombic
Space group	$P\bar{4}2_1m$	$P4_2ncm$	*I*222
a, Å	17.3590(7)	17.7189(15)	12.033(2)
b, Å	17.3590(7)	17.7189(15)	12.498(3)
c, Å	15.6801(9)	15.4728(14)	14.963(3)
α, deg	90.00	90.00	90.00
β, deg	90.00	90.00	90.00
γ, deg	90.00	90.00	90.00
V, $Å^3$	4725.0(5)	4857.8(9)	2250.4(8)
Z	4	4	2
D(calcd), g/cm^3	1.433	1.369	1.732
μ, mm^{-1}	2.255	1.375	1.531
F(000)	2074.0	2028.0	1210.0
Index ranges	$-20 \le h \le 21$<br>$-15 \le k \le 21$<br>$-19 \le l \le 14$	$-21 \le h \le 11$<br>$-21 \le k \le 18$<br>$-13 \le l \le 18$	$-15 \le h \le 13$<br>$-16 \le k \le 16$<br>$-18 \le l \le 19$
Reflections collected	16270	6776	8892
Independent reflections	4971	2196	2574
Goodness-of-fit on F^2	1.062	1.004	1.087
Final R indexes [I>=2σ (I)]	R_1 = 0.0807<br>wR_2 = 0.2148	R_1 = 0.0768<br>wR_2 = 0.2057	R_1 = 0.0521<br>wR_2 = 0.1376
CCDC	2343050	2343051	1469968

[a] CCDC number of MFM-504(Cu)-OH is 1469968, which is published in previous work.[5]

2.7 Single crystal structure of MFM-504(Cu)

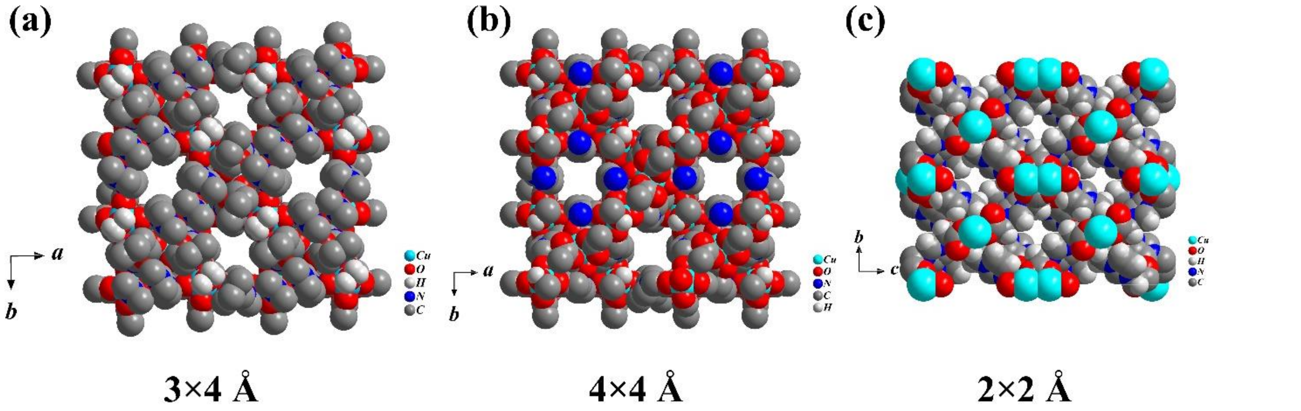

Figure S8. The space filling view of MFM-504(Cu)-DMF (a), MFM-504(Cu)-MeOH (b) and MFM-504(Cu)-OH (c) (Cu: cyan, O: red, N: dark blue, C: dark grey).

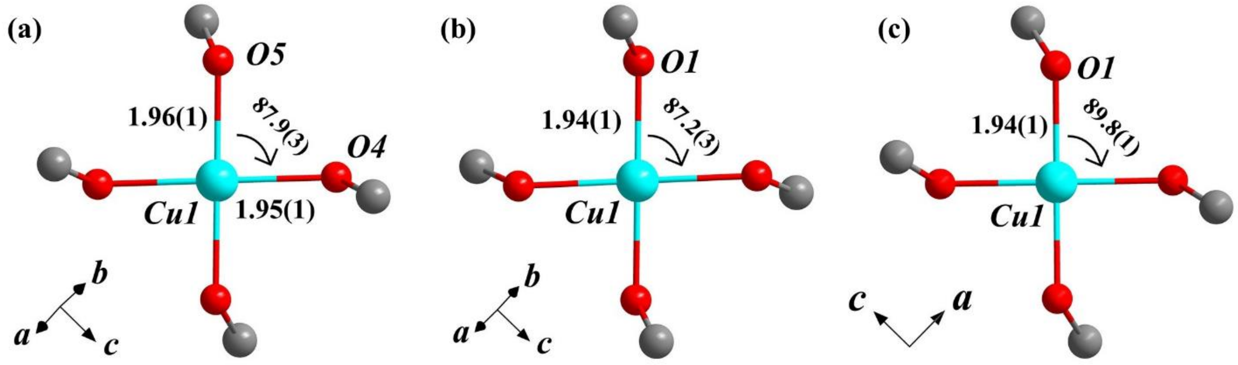

Figure S9 Views of the square planar coordination geometry of Cu1 atoms in (a) MFM-504(Cu)-DMF, (b) MFM-504(Cu)-MeOH, and (c) MFM-504(Cu)-OH (Cu: cyan, O: red, N: dark blue, C: dark grey).

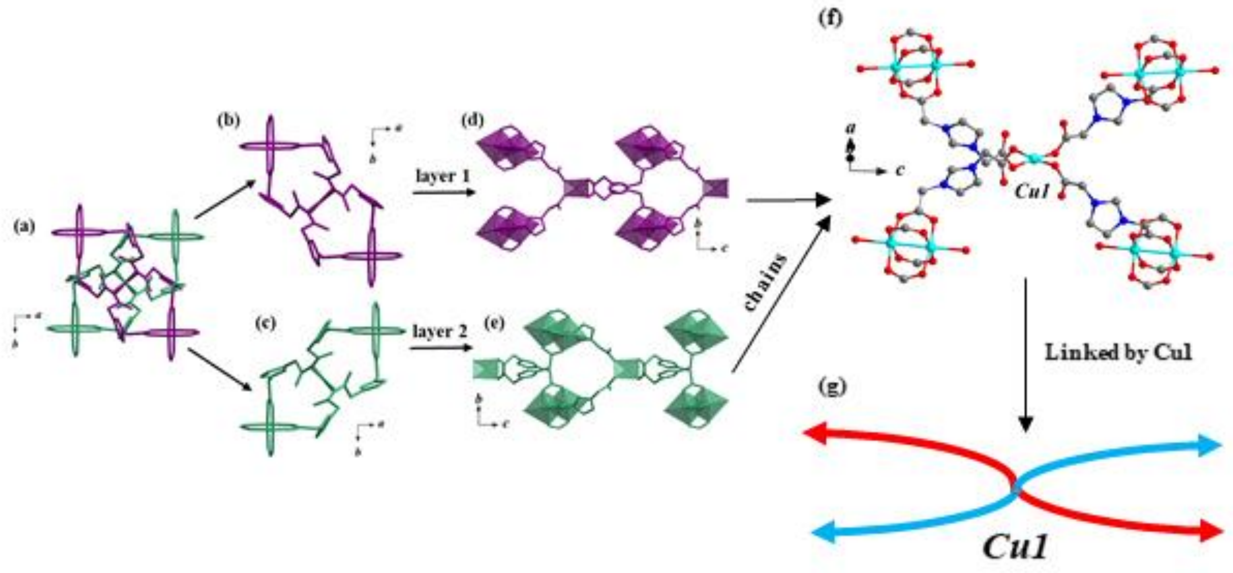

Figure S10. View of 2D layered structure of MFM-504(Cu)-DMF: (a) Interwoven 2D layered structure viewed along the *c* axis; (b)-(c) two separate layers viewed along the *c* axis; (d)-(e) two separate layers viewed along the *a* axis; (f)-(g) two chains interweaved at Cu1 (Cu: cyan, O: red, N: dark blue, C: dark grey H: light grey).

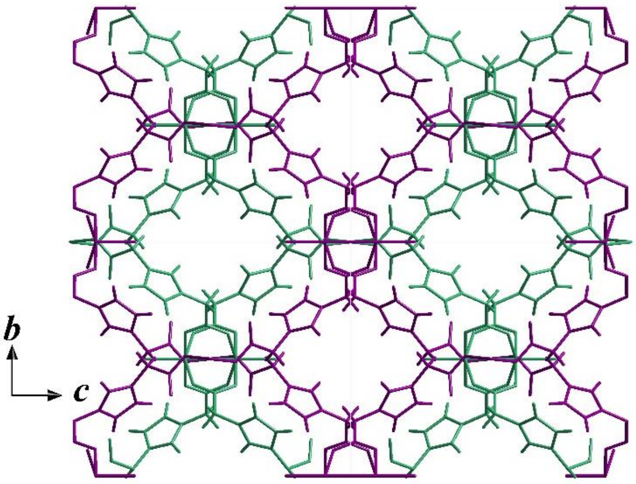

Figure S11. View of the 2D layered structure of MFM-504(Cu)-OH along the *a* axis.

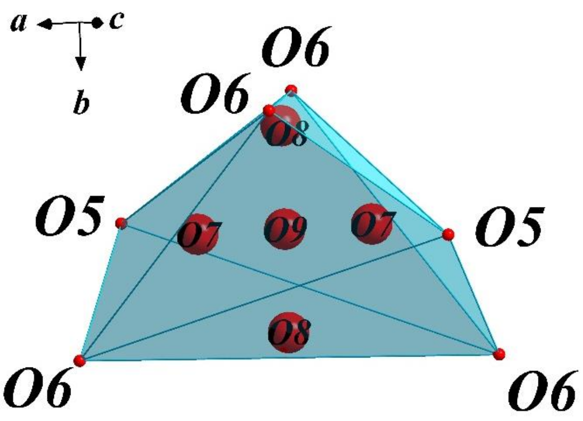

Figure S12. View of the hydrogen-bonded supra-polyhedron [O_6] in MFM-504(Cu)-OH along the *bc* plane (O: red, polyhedron: blue).

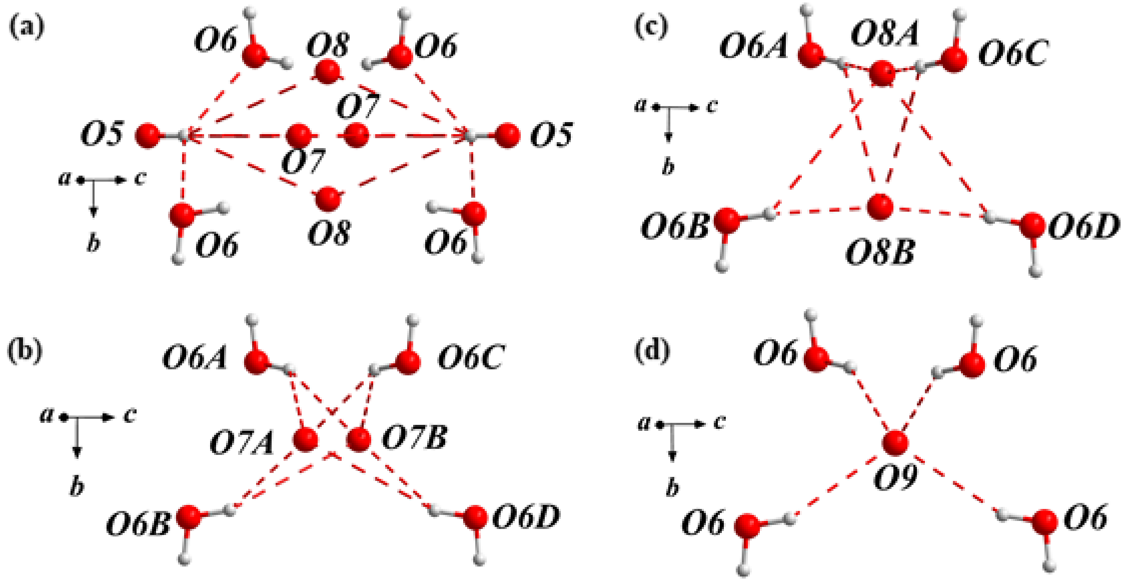

Figure S13. Views of hydrogen bonding in the MFM-504(Cu)-OH along the *bc* plane (O: red, H: light grey, hydrogen bond: red dash line).

Table S2. Summary of hydrogen bonding in MFM-504(Cu)-OH

<table>
<tr><th>D</th><th>H</th><th>A</th><th>d(D−H)/Å</th><th>d(H−A)/Å</th><th>d(D−A)/Å</th><th>D−H−A/°</th></tr>
<tr><td>O5</td><td>H5W</td><td>O6</td><td>0.83(2)</td><td>2.34(1)</td><td>2.72(1)</td><td>109.0(1)</td></tr>
<tr><td>O5</td><td>H5W</td><td>O7</td><td>0.83(2)</td><td>3.45(2)</td><td>4.23(1)</td><td>159.2(1)</td></tr>
<tr><td>O5</td><td>H5W</td><td>O8</td><td>0.83(2)</td><td>3.46(2)</td><td>4.24(1)</td><td>158.5(1)</td></tr>
<tr><td>O6</td><td>H62</td><td>O9</td><td>0.86(4)</td><td>3.56(8)</td><td>3.21(1)</td><td>152.1(31)</td></tr>
<tr><td>O6A</td><td>H62</td><td>O7A</td><td rowspan="4">0.86(4)</td><td rowspan="4">3.38(9)</td><td rowspan="4">4.03(1)</td><td rowspan="4">134.5(31)</td></tr>
<tr><td>O6B</td><td>H62</td><td>O7B</td></tr>
<tr><td>O6C</td><td>H62</td><td>O7B</td></tr>
<tr><td>O6D</td><td>H62</td><td>O7A</td></tr>
<tr><td>O6A</td><td>H62</td><td>O7B</td><td rowspan="4">0.86(4)</td><td rowspan="4">2.17(6)</td><td rowspan="4">2.94(1)</td><td rowspan="4">148.2(30)</td></tr>
<tr><td>O6B</td><td>H62</td><td>O7A</td></tr>
<tr><td>O6C</td><td>H62</td><td>O7A</td></tr>
<tr><td>O6D</td><td>H62</td><td>O7B</td></tr>
<tr><td>O6A</td><td>H62</td><td>O8A</td><td rowspan="4">0.86(4)</td><td rowspan="4">2.14(7)</td><td rowspan="4">2.93(1)</td><td rowspan="4">152.1(31)</td></tr>
<tr><td>O6B</td><td>H62</td><td>O8B</td></tr>
<tr><td>O6C</td><td>H62</td><td>O8A</td></tr>
<tr><td>O6D</td><td>H62</td><td>O8B</td></tr>
<tr><td>O6A</td><td>H62</td><td>O8B</td><td rowspan="4">0.86(4)</td><td rowspan="4">3.43(8)</td><td rowspan="4">4.06(2)</td><td rowspan="4">132.6(30)</td></tr>
<tr><td>O6B</td><td>H62</td><td>O8A</td></tr>
<tr><td>O6C</td><td>H62</td><td>O8B</td></tr>
<tr><td>O6D</td><td>H62</td><td>O8A</td></tr>
</table>

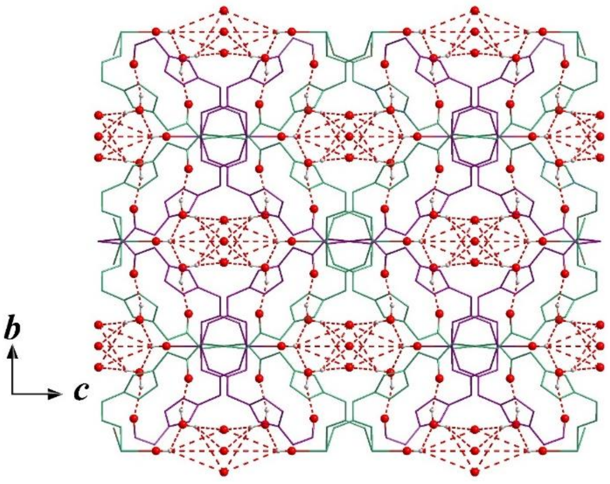

Figure S14. The extended hydrogen-bonding network for proton transfer pathway in the MFM-504(Cu)-OH in the *bc* plane (O: red, hydrogen bond: red dash line)

2.8 Proton conductivity measurement

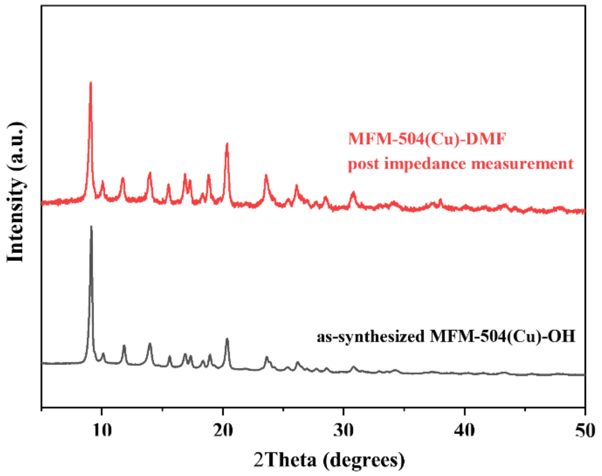

Figure S15. PXRD patterns of as-synthesised MFM-504(Cu)-OH (black) and as-synthesised MFM-504(Cu)-DMF after impedance measurements (red). The PXRD pattern of MFM-504(Cu)-DMF after impedance measurements is consistent with that of the MFM-504(Cu)-OH, which confirms the phase transition and conversion of MFM-504(Cu)-DMF to MFM-504(Cu)-OH at 25°C and 99% RH.

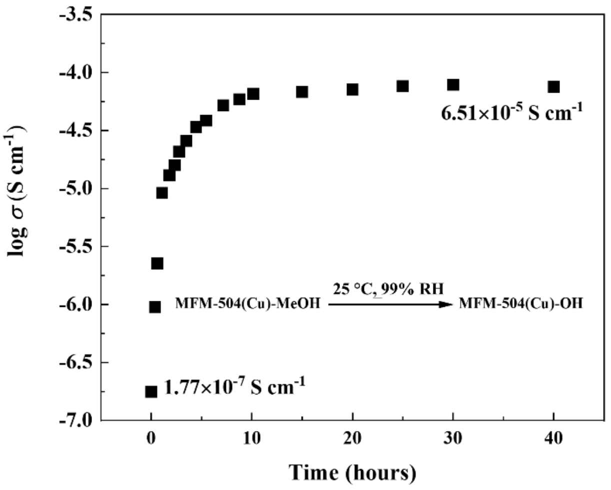

Figure S16. Time dependence of the proton conductivity during the phase transition from MFM-504(Cu)-MeOH to MFM-504(Cu)-OH at 25 °C and 99% RH

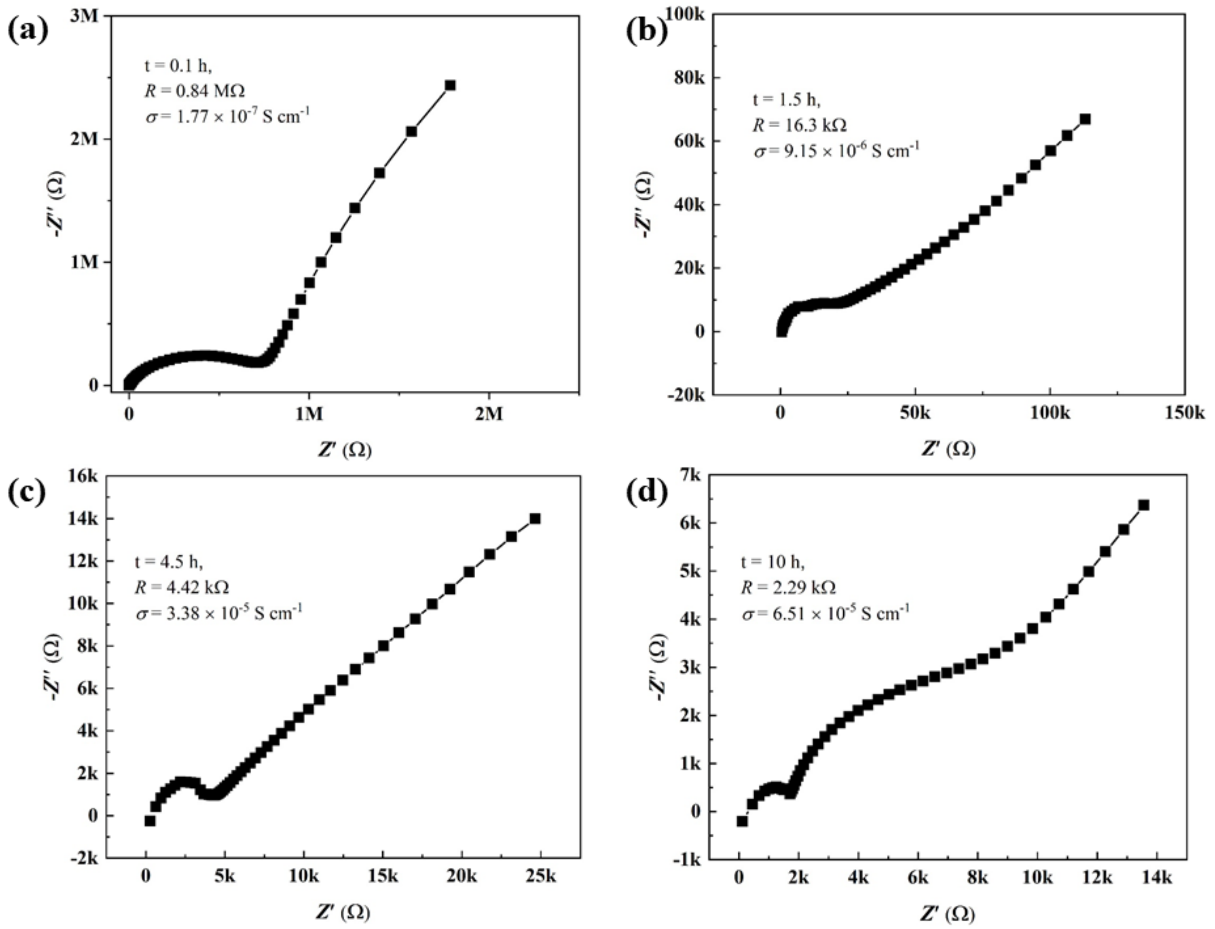

Figure S17. Nyquist plots of MFM-504(Cu)-MeOH during the phase transition.

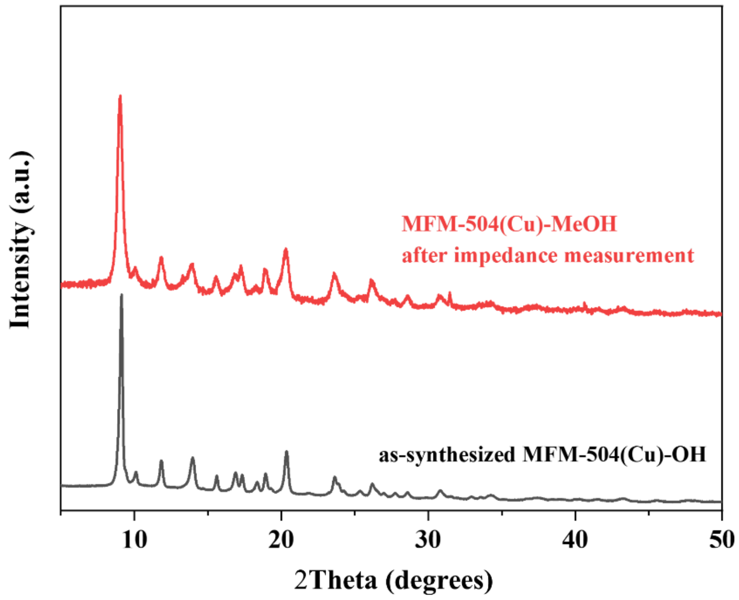

Figure S18. PXRD patterns of as-synthesised MFM-504(Cu)-OH (black) and as-synthesised MFM-504(Cu)-MeOH post the impedance measurement (red). The PXRD pattern of MFM-504(Cu)-MeOH after impedance measurements is consistent with that of MFM-504(Cu)-OH, which confirms the phase transition and conversion of MFM-504(Cu)-MeOH to MFM-504(Cu)-OH at 25°C and 99% RH.

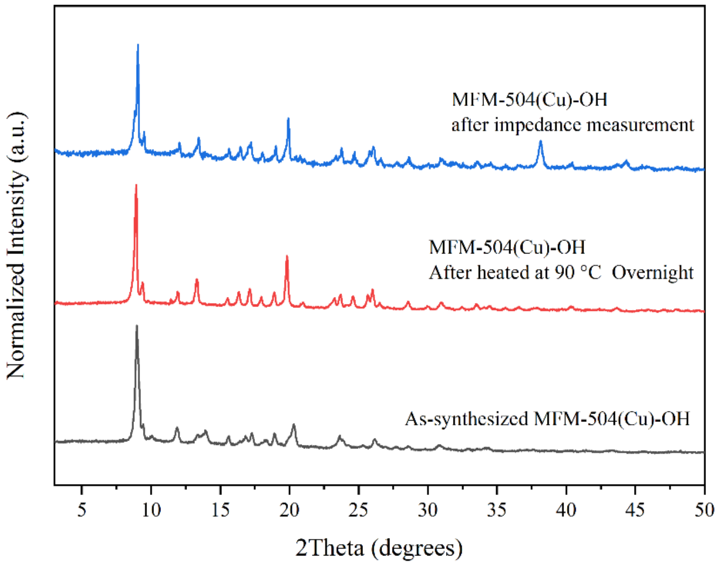

Figure S19. PXRD patterns of as-synthesised MFM-504(Cu)-OH (black) and MFM-504(Cu)-OH upon heating at 90°C overnight (red). The PXRD pattern of MFM-504(Cu)-OH after impedance measurements at 25°C and 99% RH confirms the stability of MFM-504(Cu)-OH (blue).

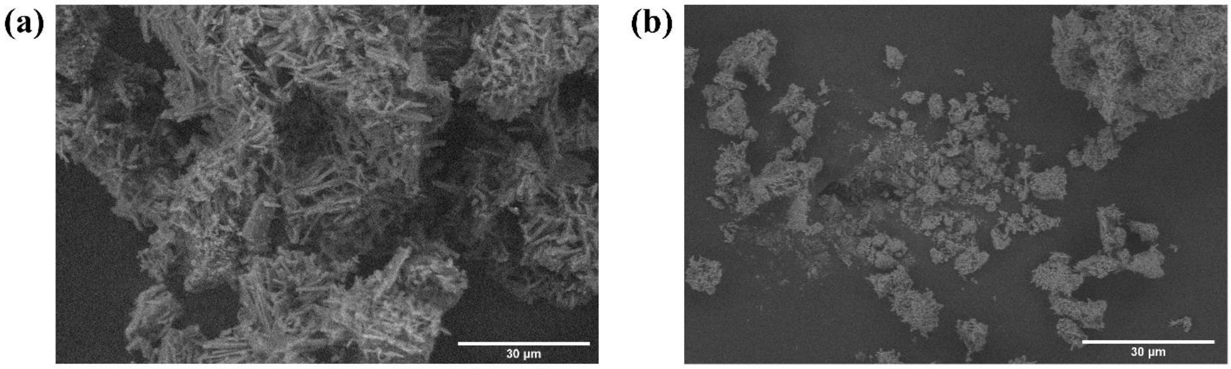

Figure S20. SEM images for MFM-504(Cu)-OH (a) before proton conductivity measurement and (b) after being ground and exposed to water during proton conductivity measurements for 24 h.

2.9 Dielectric spectra of MFM-504(Cu)-OH

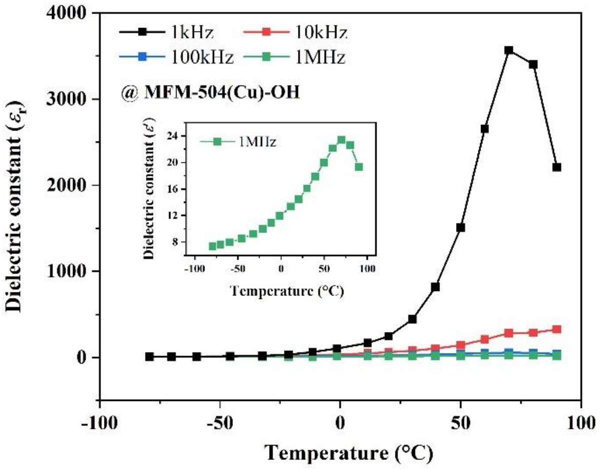

Figure S21. Various dielectric spectra of MFM-504(Cu)-OH at various frequencies from the 1kHz to 1MHz.

Because the space group *I*222 of MFM-504(Cu)-OH falls is a polar space group,[6] this makes this MOF a potential candidate as a ferroelectric material. Thus, dielectric spectra were recorded on the pellet of MFM-504(Cu)-OH as a function of temperature (Figure S21). The dielectric constant of MFM-504(Cu)-OH increases with the increase of temperature and reaches a peak value of 3500 at 1 kHz and 70 °C, and then decreases upon further increase of temperature, indicating the presence of a phase transition at 70 °C. This result is comparable to the published work that reports a similar trend of the change of the dielectric constant as a function of temperature.[7-8] For example, $[Co_2(1,4\text{-bdc})_2(dabco)]4DMF\cdot 1H_2O$ shows a colossal dielectric constant of 5000 at room temperature and 1 kHz, which is related to the presence of the water guest molecules located inside the cavities.[7] The layered perovskite $(benzylammonium)_2PbCl_4$ exhibits a sharp peak of dielectric constant at various frequencies at 165 °C (750 at 1 kHz), where the phase transition is derived from the order-disorder benzylammonium.[8] However, other characterisation is required to confirm the ferroelectric phase transition within MFM-504(Cu)-OH, such as the crystal structural determination at 70 °C, second-harmonic generation, and the electric hysteresis loop. This work is underway.

REFERENCES

(1) Barczyński, P.; Komasa, A.; Ratajczak-Sitarz, M.; Katrusiak, A.; Huczyński, A.; Brzezinski, B. Molecular Structure of 1,3-Bis(Carboxymethyl)Imidazolium Bromide and Its Betaine Form in Crystal. *J. Mol. Struct*. **2008**, *876*, 170–176.

(2) CrysAlis, C.C.D. CrysAlis Red, Xcalibur PX Software, Oxford Diffraction Ltd., Abingdon, England, **2008**.

(3) Dolomanov, O. V.; Bourhis, L. J.; Gildea, R. J.; Howard, J. A. K.; Puschmann, H. OLEX2 : A Complete Structure Solution, Refinement and Analysis Program. *J. Appl. Crystallogr* **2009**, *42*, 339–341.

(4) Yang, Y.; Urban, M. W. Self-Healing Polymeric Materials. *Chem. Soc. Rev.* **2013**, *42*, 7446-7467.

(5) Chai, X. C.; Gao, X. N.; Li, H.; Zhang, H. H.; Han, Q. P. Two Coordination Novel Polymers Based on a Flexible Ligand *N,N´*-Diacetic Acid Imidazolium. *Chin. J. Chem.* **2017**, *36*, 463-470.

(6) Zhang, H. Y.; Tang, Y. Y.; Shi, P. P.; Xiong, R. G. Toward the Targeted Design of Molecular Ferroelectrics: Modifying Molecular Symmetries and Homochirality. *Accounts of Chemical Research* **2019**, *52*, 1928–1938.

(7) Sánchez-Andújar, M.; Yáñez-Vilar, S.; Pato-Doldán, B.; Gómez-Aguirre, C.; Castro-García, S.; Señarís-Rodríguez, M. A. Apparent Colossal Dielectric Constants in Nanoporous Metal Organic Frameworks. *J. Phys. Chem. C* **2012**, *116*, 13026–13032.

(8) Liao, W. Q.; Zhang, Y.; Hu, C. L.; Mao, J. G.; Ye, H. Y.; Li, P. F.; Huang, S. D.; Xiong, R. G. A Lead-Halide Perovskite Molecular Ferroelectric Semiconductor. *Nat. Commun.* **2015**, *6*, 7338.
